# Management of insomnia in inpatient psychiatric care: a clinical practice evaluation

**DOI:** 10.1007/s00406-026-02206-0

**Published:** 2026-02-18

**Authors:** C. L. Schneider, M. Anselin, E. Hertenstein, S. Monari, C. Mikutta, M. Angelillo, H. Richard-Lepouriel, L. Soldati, M. Kosel, V. Marini, P. Cordera, C. Nissen

**Affiliations:** 1 Department of Psychiatry, Faculty of Medicine, University of Geneva, Geneva, Switzerland; 2 Department of Psychiatry, Geneva University Hospital (HUG), Geneva, Switzerland; 3 Privatklinik Meiringen, Meiringen, Switzerland; 4 Department of Psychiatry, University of Geneva, Geneva University Hospital (HUG), Rue de Lausanne 20bis, Geneva, 1201 Switzerland

**Keywords:** Sleep, Psychiatry, Insomnia, Behavioral therapy, Hypnotics, Inpatient

## Abstract

Mental disorders are the leading cause of reduced quality of life due to illness worldwide. Most patients with mental disorders report difficulties initiating or maintaining sleep (insomnia). Current guidelines recommend cognitive behavioral therapy for insomnia (CBT-I) as the first-line treatment, including for patients with comorbid mental disorders. Particularly, data on the management of insomnia in inpatient psychiatric care remain scarce. This clinical practice evaluation examined the management of insomnia in inpatient psychiatric care through two complementary approaches. The project comprised a survey on insomnia management in 80 mental health professionals and data extraction of nighttime medication from 245 inpatients. Reports from the survey indicate that 95% of mental health professionals regularly care for patients with insomnia. 56% correctly identified CBT-I and 48% incorrectly identified benzodiazepines or benzodiazepine-receptor agonists as the first-line treatment for insomnia. 65% reported that CBT-I is not or rarely offered. The main reported challenges to implementing CBT-I were limited knowledge about CBT-I, patient expectations for pharmacological treatment, and time constraints in clinical practice. Data extracted from 245 patient records indicated that 40% of the patients took benzodiazepines, 9% melatonin, 7% benzodiazepine receptor agonists, 5% clomethiazole, 4% antihistamines, and 2% phytotherapy. The results indicate that insomnia is a prevalent health problem in inpatient psychiatric care. Implementation of CBT-I or adaptations remains insufficient, and treatment with hypnotics is common. Future work is needed to further adapt and test CBT-I in inpatient psychiatric care to improve sleep and health.

## Introduction

Mental disorders are the leading cause for reduced quality of life due to illness worldwide [1]. The majority of patients with mental disorders report difficulties initiating or maintaining sleep (insomnia). According to current guidelines, cognitive behavioral therapy for insomnia (CBT-I) is the first-line treatment, including for patients with comorbid mental disorders [2–4]. However, data on the management of insomnia in psychiatric care remain scarce. This clinical practice evaluation aimed to further characterize the management of insomnia in psychiatric care.

Insomnia is a prevalent health problem. The present work focuses on inpatient psychiatric care. Although outpatient and community-based services are prioritized in current treatment concepts and guidelines, patients with acute or severe disorders may require inpatient care. A recent multicenter study conducted by the Sleep section of the German Association for Psychiatry, Psychotherapy and Psychosomatics (DGPPN) on the prevalence of insomnia in inpatient psychiatric care based on comprehensive interviews showed that 30% of patients across diagnostic entities fulfill diagnostic criteria for comorbid insomnia disorder according to Diagnostic and Statistical Manual of Mental Disorder criteria [5] and that additionally 40% of patients report insomnia symptoms (in preparation for publication). However, the management of insomnia in inpatient psychiatric care, including the perspectives of mental health professionals and the choice of treatment pathways, remains insufficiently evaluated.

According to current guidelines, the first-line treatment for insomnia is CBT-I and this recommendation extends to patients with mental disorders [2–4]. CBT-I not only improves sleep but also has the potential to improve other mental health outcomes [6]. However, few studies have started to adapt CBT-I to the needs of severely ill patients and health care teams in inpatient psychiatric care (for instance [7, 8],; for systematic review please refer to [9]). Still to date, CBT-I is not systematically implemented into routine clinical care. Potential reasons for this know-do gap, including knowledge on treatment guidelines and challenges to implementation, need to be further characterized. Further elucidating the knowledge and perspectives of mental health professionals and potential challenges appears important to guide future training programs, resource allocation and steps to implementation.

Despite strong evidence for the efficacy of CBT-I and its recommendation in clinical guidelines, hypnotics remain a common treatment for insomnia in inpatient psychiatric care [10–12]. Studies examining pharmacological treatment of insomnia in general hospital settings demonstrate occasional to frequent use of hypnosedative medication [13, 14]. Analyses of the prescription of hypnotics in psychiatric outpatient care reveal high rates of prescription [15]. However, comprehensive data on prescription patterns in inpatient psychiatric care remain limited. Prior work primarily reported on the total dosage of hypnotics [10] or benzodiazepines and benzodiazepine receptor agonists (BZRA) [11] and did not provide a more complete picture of prescription patterns of hypnosedative substances. Therefore, further assessment of hypnotic medication use in inpatient psychiatry is needed.

This clinical practice evaluation examined the management of insomnia in inpatient psychiatric care through two complementary approaches. First, a survey of mental health professionals assessed the frequency with which they care for patients with insomnia, their familiarity with first-line treatment recommendations, the extent to which CBT-I is offered, and potential barriers to its implementation. Second, data extraction from patient records evaluated patterns of hypnotic nighttime medication intake.

## Methods

### Overview

The study was conducted at the Department of Psychiatry of the Geneva University Hospital (HUG), Switzerland. The current project focused on inpatient psychiatric care, which encompasses the work of 420 mental-health professionals, including physicians, nurses, psychologists, and others, providing care for 250 adult inpatients with mental disorders across all diagnostic entities in the Canton of Geneva (for a total of around 3’500 patients per year).

This clinical practice evaluation comprised two complementary parts, a survey on insomnia management of mental health professionals and a data extraction of nighttime medication.

The cantonal ethics committee (Commission cantonale d’éthique de la recherche sur l’être humain, CCER) declared that the project is considered a quality assurance project that does not fall under the Human Research Act (Req-2025-00976). The study protocol and survey questions were submitted to and approved by the local ethics committee of the Geneva University Hospital (HUG) (Conseil d’évaluation des études ne relevant pas de la Commission d’éthique de la recherche, CEENCER, protocole n° 2025-05). Data was collected via Microsoft Forms following data protection guidelines.

### Part 1: survey—insomnia treatment

#### Sample

The survey was addressed to mental health professionals (including but not limited to physicians, psychologists, and nurses) in the inpatient setting of the Department of Psychiatry, Geneva University Hospital (HUG). The invitation was sent via e-mail to 318 mental health professionals who were present at the time of the survey (excluding those absent due to training, vacation, illness, or other leave).

#### Survey

This clinical practice evaluation centered on key outcomes reflecting the management of insomnia in inpatient psychiatric care. The online survey contained 8 items: (1) how often mental health professionals care for patients with insomnia symptoms, (2) guideline knowledge on the first-line treatment of insomnia disorder, (3) how often CBT-I is offered in inpatient care, and (4) the challenges of implementation. These questions were followed by (5) participants’ professional group, (6) length of their employment, (7) age, and (8) gender. Responses to questions 1 and 3 were measured on a 5-point Likert scale commonly recommended as a balanced option between measurement precision, respondent usability, and statistical robustness. Questions 2 and 4 were answered through multiple-response multiple-choice questions. Questions 5 to 8 were answered through single-response multiple-choice questions.

The survey was developed by the research team and pretested with health professionals to ensure clarity. The survey was hosted on Microsoft Forms in French and took approximately 4 min to complete, respecting the limited time resources in inpatient care.

Mental health professionals first received an announcement and were subsequently invited to participate via email one week later. The survey remained open between May and June 2025 (24 days). Reminder emails were sent out in week 1 and 3 after the start of data collection.

### Part 2: data extraction—nighttime medication

#### Sample

The sample consisted of all inpatients in the Department of Psychiatry, Geneva University Hospital (HUG) on an index day.

#### Data extraction

A retrospective review of electronic patient records was conducted for all inpatients on a randomly selected index day in September 2025. The use of a randomized index day is a robust method for clinical practice evaluations, as it helps minimize selection bias, although it does not account for temporal variability in medication usage. Data was extracted from electronic medical records using a standardized form encompassing different variables. These variables included date of admission, legal status, hospital ward, demographic and clinical characteristics (e.g., age, sex, primary diagnosis, insomnia diagnosis), and documented intake of hypnosedative medications on the evening or night prior to the index day. The following medications were classified as *probable hypnotic medication*: Benzodiazepines (e.g., midazolam, oxazepam, lorazepam, alprazolam, clonazepam), benzodiazepine receptor agonists (BZRA) (zolpidem, zopiclone, or zaleplon), antihistamines (e.g., hydroxyzine), clomethiazole, daridorexant, melatonin, phytotherapy (e.g., valverde or other herbal remedies), and cannabis preparations. The following medications were classified as *potential* hypnotic medication: sedative antidepressants (e.g., trazodone, trimipramine, mirtazapine), sedative antipsychotics (e.g., quetiapine, levomepromazine, chlorprothixene, clotiapine, olanzapine), and pregabalin. The underlying rationale for the prescription was not evaluated in this project. Consequently, our interpretation centered on *probable* hypnotic medication given that these compounds are frequently prescribed for the management of insomnia. In contrast, compounds classified as *potential* hypnotic medication are frequently prescribed for other symptoms in psychiatric care, precluding further interpretation regarding their potential prescription for insomnia symptoms. Data collection was conducted using Microsoft Forms.

### Data analysis

All analyses were performed using Microsoft Forms and Excel. For Part 1, descriptive statistics (means, standard deviations, frequencies, and percentages) were calculated as appropriate. For Part 2, descriptive analyses summarized the frequency of medications.

## Results

### Part 1: survey—insomnia treatment

#### Participation

**Fig. 1:**
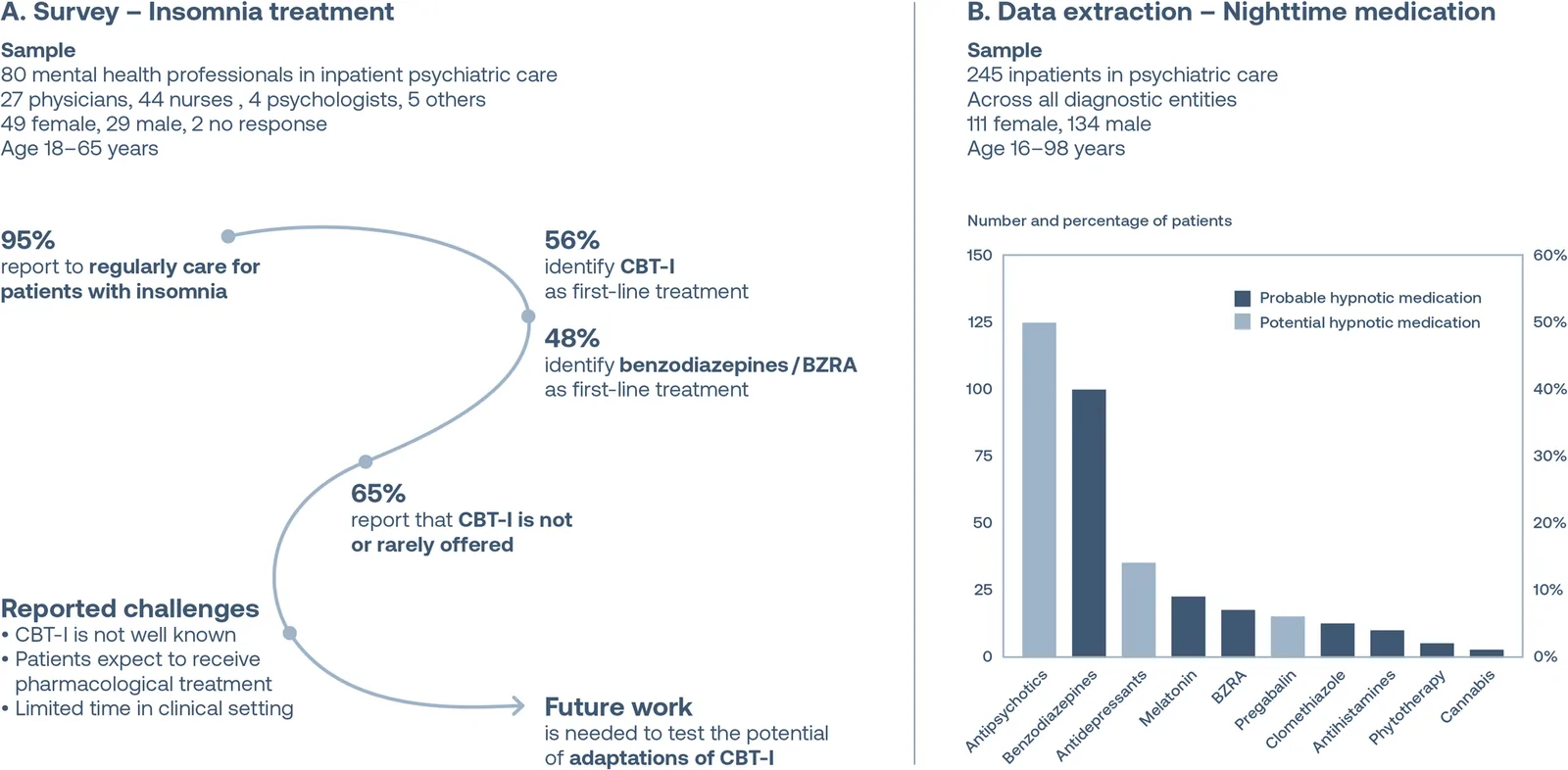
Management of insomnia in inpatient psychiatry. Panel (**A**) Survey—insomnia treatment. The panel depicts a description of the sample of mental health professionals in inpatient psychiatric care and the major results regarding care for patients with insomnia, guideline knowledge, implementation of CBT-I, and reported challenges. Panel (**B**) Data extraction—nighttime medication. The panel depicts a description of the sample of inpatients in psychiatric care and the number (left y-axis) and percentage (right y-axis) of patients receiving probable or potential hypnotic medication

Of the 318 mental health professionals contacted, 80 completed the survey (response rate of 25.2%). Participants included 27 physicians, 44 nurses, 4 psychologists, and 5 others. The age range was 18 to 65 years. Forty-nine of the participants indicated that they were female, 29 indicated that they were male, and 2 chose not to respond (Fig. 1, Panel A). About half of the sample reported having worked in their current workplace for more than ten years.

#### Reports from mental health professionals

The survey centered on four major outcomes, as depicted in Fig. 1, Panel A.

First, health professionals reported regularly caring for patients with insomnia symptoms. More specifically, 95.0% (*n* = 76) reported regular care as defined by indicating common, often, very often, and nearly all the time on the Likert scale. Of 27 physicians, 3.7% (*n* = 1) indicated to care for patients with insomnia symptoms nearly all the time, 55.6% (*n* = 15) very often, 29.6% (*n* = 8) often, 11.1% (*n* = 3) commonly, and none reported to rarely care for patients with insomnia symptoms. Of 44 nurses, 4.6% (*n* = 2) indicated to care for patients with insomnia symptoms nearly all the time, 34.1% (*n* = 15) very often, 27.3% (*n* = 12) often, 31.8% (*n* = 14) commonly, and 2.3% (*n* = 1) reported to rarely care for patients with insomnia symptoms. Of 4 psychologists, 1 indicated to care for patients with insomnia symptoms nearly all the time, 1 very often, and 2 indicated to commonly care for patients with insomnia symptoms. Of 5 other mental health professionals, 2 indicated to commonly care for patients with insomnia symptoms and 3 reported to rarely care for patients with insomnia symptoms.

Second, 56.3% (*n* = 45) of the participants (18 physicians, 23 nurses, 3 psychologists, and 1 other) identified CBT-I as the first-line treatment for insomnia disorder. 47.5% (*n* = 38) of the participants (9 physicians, 25 nurses, 1 psychologist, 3 others) identified benzodiazepines or benzodiazepine-receptor agonists as the first-line treatment for insomnia disorder. Further treatment options were identified as first-line treatment in the following order (multiple-response multiple-choice question): Sleep hygiene (*n* = 60, 75%), melatonin (*n* = 42, 52.5%), antidepressants (*n* = 33, 41.3%), antipsychotics (*n* = 16, 20.9%), phytotherapy (*n* = 13, 16.3%), antihistamines (*n* = 13, 16.3%), other psychological therapies for insomnia (*n* = 5, 6.3%) and daridorexant (*n* = 2, 2.5%). No alternative treatments were identified as first-line treatments.

Third, 65.0% (*n* = 52) of the mental health professionals reported that CBT-I is not or rarely offered as a treatment for patients with insomnia. Of 27 physicians, 74.1% (*n* = 20) reported that CBT-I is rarely offered to patients with insomnia, 18.5% (*n* = 5) commonly, 3.7% (*n* = 1) very often, and 3.7% (*n* = 1) indicated that CBT-I is offered to patients nearly all the time. Of 44 nurses, 56.8% (*n* = 25) reported that CBT-I is rarely offered to patients with insomnia, 25.0% (*n* = 11) commonly, 4.5% (*n* = 2) often, 4.5% (*n* = 2) very often, and 4.5% (*n* = 2) indicated that CBT-I is offered to patients nearly all the time. Two nurses indicated that no response was possible. All psychologists (*n* = 4) reported that CBT-I is rarely offered to patients with insomnia. Of 5 other mental health professionals, 3 indicated that CBT-I is rarely offered to patients with insomnia and 2 indicated that no response was possible.

Fourth and lastly, participants reported challenges of implementation in the following order. The main reason as to why CBT-I is not systematically implemented in inpatient psychiatric care was that CBT-I is not well known (reported 54 times), followed by the challenge that patients expect to receive pharmacological treatment (reported 44 times), that there is limited time in the clinical setting (reported 35 times), that other problems often appear more important and urgent (reported 24 times), and that patients cannot implement CBT-I (reported 17 times).

### Part 2: data extraction—nighttime medication

Medical records of all inpatients at the Department on an index day in September 2025 were reviewed for hypnotic medication intake on the evening and night prior to the index day (Fig. 1, Panel B). Of 250 patient records, 245 were included in the analysis. Five records were excluded as the admission date coincided with the data collection date, preventing data extraction of nighttime medication administered on the preceding evening or night. The sample included 111 female and 134 male patients with an age range of 16 to 98 years (mean age 53.1 years, SD = 22.1). The hospital stay ranged between 1 and 2823 days. The Health of the Nation Outcome Scales (HoNOS) [16] score was available for 141 patients (mean score 26.6, SD 8.6), reflecting severe illness. 145 patients (59.2%) were under involuntary commitment. The sample included patients across all diagnostic entities according to ICD-10 criteria [17] (main diagnoses: *n* = 53: F0, *n* = 22: F1, *n* = 57: F2, *n* = 51: F3, *n* = 6: F4, *n* = 5: F5, *n* = 14: F6, *n* = 13: F7, *n* = 7: F8, *n* = 0: F9). For 17 patients, no diagnosis was documented, most likely due to recent admission and pending diagnosis. Only one out of 245 patients had a documented diagnosis of insomnia disorder.

Data extraction (Fig. 1, Panel B) demonstrated that sedative antipsychotics were most frequently prescribed (*n* = 122, 49.8%), followed by benzodiazepines (*n* = 98, 40.0%) and sedative antidepressants (*n* = 34, 13.9%). Less commonly prescribed were melatonin (*n* = 23, 9.4%), BZRA (*n* = 18, 7.4%), pregabalin (*n* = 15, 6.1%), clomethiazole (*n* = 13, 5.3%), antihistamines (*n* = 9, 3.7%), phytotherapy (*n* = 5, 2.0%), and cannabis preparations (*n* = 3, 1.2%). Daridorexant was not prescribed. It is important to note that sedative antipsychotics and antidepressants as well as pregabalin were categorized as *potential* hypnotic medications, while all other medications were classified as *probable* hypnotic medications. A substantial proportion of patients received polypharmacy with hypnotic medications (*n* = 103, 42.0%). 58 patients (23.7%) did not take any nighttime medication. It is to note that we did not distinguish between non-prescription and non-adherence.

To examine whether benzodiazepines were administered as short- or long-term treatment, we analyzed the intake of benzodiazepines relative to the duration of the inpatient stay. Among patients with an inpatient stay of up to 3 days (*n* = 20), 7 (35.0%) took benzodiazepines. Among patients with an inpatient stay of 4 to 10 days (*n* = 35), 17 (48.6%) took benzodiazepines. Among patients with an inpatient stay of 11 to 30 days (*n* = 52), *25* (48.1%) took benzodiazepines. Among patients with an inpatient stay exceeding 30 days (*n* = 138), 49 (35.5%) took benzodiazepines. Out of 145 patients with involuntary commitment, 63 took benzodiazepines (43.4%). Out of 100 patients with voluntary commitment, 35 took benzodiazepines (35.0%). A chi-square test revealed no significant difference in the intake of benzodiazepines between the groups (*χ*^*2*^ = 1.4, *df*: 1, *p* = 0.232). These findings indicate that benzodiazepine intake was not limited to acute treatment or involuntary status.

## Discussion

This clinical practice evaluation indicates that almost all mental health professionals (95%) in the investigated inpatient psychiatry setting care for patients with insomnia symptoms. Slightly more than half (56%) correctly identified cognitive behavioral therapy for insomnia (CBT-I), and about half (48%) incorrectly identified benzodiazepines or benzodiazepine receptor agonists (BZRA) as the first-line treatment for insomnia disorder according to guidelines. Reports indicate that CBT-I is not systematically implemented in inpatient psychiatric care, and challenges were identified. Data extracted from patient records show that intake of sedative or hypnotic nighttime medication is common, with 40% of patients taking benzodiazepines. These results provide direct evidence that implementation of CBT-I in inpatient psychiatric care is insufficient, and treatment with hypnotics is frequent.

### Survey—insomnia treatment

Findings from our survey provide new data on the perspective of mental health professionals in inpatient psychiatric care.

Close to all participants (95%) reported that they regularly care for patients with insomnia symptoms. This adds a health professional perspective to recent prevalence data showing that 30% of inpatients in psychiatric care fulfill diagnostic criteria for comorbid insomnia disorder (DSM-5) and additional 40% of patients report insomnia symptoms (in preparation for publication). These observations provide converging evidence that insomnia is a frequent challenge in clinical care.

Slightly more than half of the mental health professionals (56%) correctly identified CBT-I as the first-line treatment for insomnia disorder, indicating that about half of the participants did not identify CBT-I as first-line treatment. Close to half of participants (48%) incorrectly identified benzodiazepines or BZRAs as the first-line treatment for insomnia disorder. When comparing guideline knowledge per professional group, physicians represented the best-informed group. To our knowledge, we present the first assessment of guideline knowledge about insomnia treatment from mental health professionals in inpatient psychiatric care. The results illustrate the need for further information and training regarding guideline recommendations on insomnia treatment.

The majority of the participants (65%) reported that CBT-I is not or rarely offered to patients with insomnia. These reports complement our systematic review that indicated a paucity of studies on CBT-I or adaptations in inpatient psychiatry [9] by a professionals’ perspective on routine clinical care. To our knowledge, this is the first report providing data from mental health professionals in inpatient psychiatry on how often patients with insomnia receive first-line treatment.

Participants were asked to identify challenges preventing the implementation of CBT-I in the inpatient setting. The main reasons were insufficient knowledge about CBT-I, followed by patients’ expectations to receive pharmacological treatment, time constraints in the clinical setting, other urgent problems, and patients’ inability to implement CBT-I. Interestingly, doubts about feasibility of CBT-I in the population of severely ill patients was chosen least frequently. Our recent review shows that adaptations of CBT-I for severely ill patients and limited resources in inpatient psychiatric care are feasible [9].

The results of the survey should be interpreted with some caution. Despite the anonymous character of the survey, a response bias toward socially desirable answers cannot be excluded, given that it was administered by clinicians and researchers known for a focus on sleep, insomnia and CBT-I. Such a bias would be expected to favor answers towards CBT-I; still, close to half of mental health professionals did not identify CBT-I as the first-line treatment and indicated low implementation. A limitation is that out of 318 invited mental health professionals, 80 completed the survey. The response rate of 25.2% is within the expected range for survey data in routine clinical settings [18]. If selection bias occurred, it would likely favor participation of those with a greater interest in insomnia and potentially guideline knowledge; thus, guideline knowledge and the implementation of CBT-I might be rather worse than better with extended participation.

### Data extraction—nighttime medication

Data extraction revealed a frequent intake of nighttime sedative medication in the investigated inpatient psychiatry setting, with benzodiazepines among the most prescribed agents (40% of patients). Notably, benzodiazepine intake was not limited to acute treatment or involuntary status but was often continued throughout the first month of hospitalization and beyond, despite the established risks of tolerance, dependence, and the lack of indications for long-term use. This pattern is particularly concerning, as pharmacological treatment not only carries safety risks but also fosters expectations among patients and clinicians that medication is necessary for sleep, thereby hindering the uptake of behavioral treatment. Long-term remission rates of CBT-I combined with medication are worse than those for CBT-I alone and medication has the potential to diminish positive treatment effects of CBT-I [19, 20]. CBT-I compared to pharmacological treatment is more effective in the long term and reduces the prescription of hypnotics [21, 22]. Strategies to foster implementation of CBT-I or adaptations and de-implementation of routine prescriptions of benzodiazepines are needed.

Other common medications likely prescribed for hypnotic action include melatonin and BZRAs. Other substances, including antihistamines and phytotherapy, were less frequently prescribed. The newer orexin receptor antagonist daridorexant was not prescribed. Further identified medications included sedative antipsychotics and antidepressants. Since the indication remained unclear in the context of complex comorbidity, including severe psychotic and affective disorders, these latter medications were classified as *potential* hypnotic medications.

Some limitations of the data extraction of medication need to be addressed. First, data extraction was restricted to a single index day, preventing conclusions about longer-term patterns. Moreover, assessing the indication for prescription was beyond the scope of the current project. Therefore, it cannot be determined whether identified medications were prescribed for insomnia or for comorbid conditions. Of note, only one out of 245 patients had a documented diagnosis of insomnia disorder.

## General limitations, strengths and conclusions

In addition to the specific limitations discussed above, the present project has other contextual constraints. Although the Department of Psychiatry of the Geneva University Hospital (HUG) is partly representative of other large centers, service structures and mandates vary substantially across regions, healthcare systems, and countries. Geneva promotes an outpatient-oriented care model and provides approximately 50 inpatient psychiatric beds per 100’000 inhabitants (250 beds for a population of 500’000), which is below the European average of about 73 beds per 100’000 inhabitants and far lower than in some larger metropolitan areas. As a result, inpatient services in Geneva provide primarily care for patients with more acute and severe conditions. Moreover, differences in professional training, such as the integration of psychotherapy into psychiatry residency programs in some countries (e.g., Switzerland and Germany) but not others (e.g., France), limit the generalizability of our findings to other healthcare contexts. Nonetheless, both clinical experience and our systematic review [9] suggest that the implementation of adaptations of CBT-I in inpatient psychiatric care is unlikely to be substantially higher in other regions or countries.

The current project has strengths. It provides, to our knowledge, the first practice evaluation in inpatient psychiatric care. Given that an estimate of 2.5–3 million people are treated in inpatient psychiatry in 1 year in the European Union (EU) – estimate based on an average of 73 psychiatric hospital beds/100’000 inhabitants [23] and an average length of stay in the hospital of 39.4 days [24] – it is plausible to assume that substantive health care efforts and associated costs are allocated to sleep and insomnia, although health care estimates are, to our knowledge, not available. From our experience, discussions on sleep and insomnia are frequent in hospital settings, but often not well structured. The current project indicates that mental health professionals very frequently care for patients with comorbid insomnia. Knowledge about and implementation of CBT-I is insufficient, and treatment with hypnotics, including benzodiazepines, is frequent. In line with international recommendations, future work is needed to further adapt and test CBT-I in inpatient psychiatric care to promote the systematic uptake of evidence-based management into routine practice. Some developments indicate that adapting CBT-I to the needs of patients with severe mental disorders and health care teams in a complex medical setting is feasible [7, 8]. For instance, our SLEEPexpert program is a fully manualized [25] and freely accessible (www.sleepexpert.ch) project that can be implemented in inpatient psychiatric care. Together, the findings of the current evaluation represent a call to action. Future work is needed to test the potential of adaptations of CBT-I in inpatient psychiatric care to improve sleep and mental health.

## Data Availability

Data is available upon request to the corresponding author.
